# Short-term high-fat diet alters the mouse brain magnetic resonance imaging parameters consistently with neuroinflammation on males and metabolic rearrangements on females. A pre-clinical study with an optimized selection of linear mixed-effects models

**DOI:** 10.3389/fnins.2022.1025108

**Published:** 2022-11-24

**Authors:** Basilio Willem Campillo, David Galguera, Sebastian Cerdan, Pilar López-Larrubia, Blanca Lizarbe

**Affiliations:** ^1^Instituto de Investigaciones Biomédicas Alberto Sols (IIBm), Consejo Superior de Investigaciones Científicas-Universidad Autónoma de Madrid (CSIC-UAM), Madrid, Spain; ^2^Departamento de Bioquímica, Universidad Autónoma de Madrid (UAM), Instituto de Investigaciones Biomédicas Alberto Sols (CSIC-UAM), Madrid, Spain

**Keywords:** high-fat diet, sexual dimorphism, MRI, obesity, inflammation, brain, HRMAS, mixed-effects models

## Abstract

**Introduction:**

High-fat diet (HFD) consumption is known to trigger an inflammatory response in the brain that prompts the dysregulation of energy balance, leads to insulin and leptin resistance, and ultimately obesity. Obesity, at the same, has been related to cerebral magnetic resonance imaging (MRI) alterations, but the onset of HFD-induced neuroinflammation, however, has been principally reported on male rodents and by *ex vivo* methods, with the effects on females and the origin of MRI changes remaining unassessed.

**Methods:**

We characterized the onset and evolution of obesity on male and female mice during standard or HFD administration by physiological markers and multiparametric MRI on four cerebral regions involved in appetite regulation and energy homeostasis. We investigated the effects of diet, time under diet, brain region and sex by identifying their significant contributions to sequential linear mixed-effects models, and obtained their regional neurochemical profiles by high-resolution magic angle spinning spectroscopy.

**Results:**

Male mice developed an obese phenotype paralleled by fast increases in magnetization transfer ratio values, while females delayed the obesity progress and showed no MRI-signs of cerebral inflammation, but larger metabolic rearrangements on the neurochemical profile.

**Discussion:**

Our study reveals early MRI-detectable changes compatible with the development of HFD-induced cerebral cytotoxic inflammation on males but suggest the existence of compensatory metabolic adaptations on females that preclude the corresponding detection of MRI alterations.

## Introduction

Obesity is a chronic disease associated with several comorbidities such as type 2 diabetes, cardiovascular disease and hypertension, some types of cancer, and an increased risk of developing neurodegenerative disorders (Frisardi et al., 2010; Apovian, 2016). Affecting an unceasingly increasing population number -about 11% of men and 15% of women- this pandemic syndrome was the 5^th^ leading cause of worldwide death in 2016 (Smith and Smith, 2016). Sex differences in glucose homeostasis, fatty acid content and distribution, and the role of sex hormones, among other effects, lead to divergences in obesity and diabetes development between men and women (Mauvais-Jarvis, 2017). Despite such important differences, many of the pre-clinical investigations in the field of metabolic disorders are still based in male models exclusively (Zucker and Beery, 2010; Mauvais-Jarvis et al., 2017).

During obesity, the accumulation of elevated fat stores triggers a global, and permanent, inflammatory status that eventually leads to insulin and leptin resistance, paving the way to obesity-related co-morbidities (Ellulu et al., 2017). Additionally to systemic inflammation, pre-clinical models of diet-induced obesity (DIO) have demonstrated that high-fat diet (HFD) intake activates a localized inflammatory profile in the hypothalamus (De Souza et al., 2005; Thaler et al., 2012), the main cerebral region controlling energy balance and hunger (Timper and Bruning, 2017). Complex interactions between the immune and the nervous system regulate the function of hypothalamic neurons after HFD intake, to promote food intake and obesity (Folick et al., 2022; Kim, 2022). Particularly, inflammatory signaling is initiated by the large amounts of long-chain saturated fatty acids (SFAs) contained in the HFDs, which cross the blood-brain barrier (BBB), bind to the peptide proopiomelanocortin (POMC) neurons in the arcuate nucleus (ARC) of the hypothalamus, and induce the expression of pro-inflammatory genes (Zhang et al., 2008; Posey et al., 2009). Consequently, glial cells undergo morphological, physiological, and functional alterations enabling the HFD-induced inflammatory process against the excess of SFAs (Valdearcos et al., 2014, 2018; Ramalho et al., 2018; García-Cáceres et al., 2019). Particularly, astrocytes develop a reactive phenotype in the ARC detected immunohistochemically 24 h after HFD intake (Horvath et al., 2010; Thaler et al., 2012; Buckman et al., 2015), release inflammatory cytokines (Gupta et al., 2012), and, in response to elevated leptin levels, trigger microvascular remodeling within the hypothalamus (Yi et al., 2012; Gruber et al., 2021). Changes have been even reported at a meal-scale basis, including a glial retraction on POMC neurons that increases POMC activity after a standard, but not a high-fat, meal (Nuzzaci et al., 2020), and augmented gene expression of astrocyte and microglial markers only a few hours after a high-food consumption (Cansell et al., 2021). Interestingly, pro-inflammatory gene expression, and cellular gliosis, can be detected during the early response to fat diets (from days 1 to 14), disappearing 3 weeks after, and reappearing after long-term consumption (Thaler et al., 2012), which is in agreement with an adaptative period in which the excessive energy intake is being regulated (Ziotopoulou et al., 2000; Benani et al., 2012). Notably, all these events, have been described principally using pre-clinical male models of DIO, and several studies have challenged the existence of such hypothalamic inflammation in females, with estrogens eliciting protective roles (Morselli et al., 2014, 2016), and a higher density of POMC neurons inducing anorexigenic effects (Wang et al., 2018). A recent study in humans, however, found very limited sex differences in microglial morphology or density, in different stages of the human lifespan, in contrast with the previous rodent literature (Menassa et al., 2022).

Beyond the hypothalamus, further cerebral structures participate in the control of food intake and exert significant roles in favoring obesity development. In mammals, the homeostatic system interacts with motivational and rewarding behaviors via the mesocorticolimbic complex (MC) and reward centers (RC), respectively (Ferrario et al., 2016). MC structures include abundant dopamine projections from the ventral tegmental area to the prefrontal cortex, amygdala, hippocampus, nucleus accumbens (NAc), and infralimbic area (ILA), correlating potential appetite stimuli to associated rewards, thus creating motivational connections (Berridge, 2009). RCs, on the other hand, include regions from the orbitofrontal cortex, amygdala, and NAc, and grant food with its pleasurable properties (Saper et al., 2002). Interestingly, some of the implicated motivational and reward centers, also express inflammatory signals during obesity development (Cazettes et al., 2011). Particularly, in mice, short-term HFD administration has been reported to trigger hippocampal dysfunction associated with BBB disruption and neuroinflammation, as well as a progressive breakdown of synaptic and metabolic function (de Paula et al., 2021), and metabolic rearrangements have been reported in the mouse hippocampus after a few weeks of consumption (Lizarbe et al., 2018; Garcia-Serrano et al., 2022).

Magnetic resonance imaging (MRI), spectroscopy (MRS), and high-resolution magic angle (HRMAS) MRS, have already contributed significantly to revealing central aspects in the cerebral control of obesity onset and development. In particular, these methods have shown that obesity alters cerebral function and structure in humans and that HFDs induce microstructural and functional changes in the mouse brain (Lizarbe et al., 2020). For example, using T_2_ weighted imaging (T_2_WI), a significant rise in hypothalamic T_2_ values of 21-weeks HFD mice was detected (Lee et al., 2013), with the observed T_2_ increments correlating positively with astrocytosis and microgliosis. In humans, positive correlations between relative T_2_ signal intensities on the left medio-basal hypothalamus and the body mass index were reported, suggesting a similar response (Thaler et al., 2012). Using diffusion weighting imaging (DWI), we identified changes in the mouse hypothalamus consistent with hunger-induced cellular swelling (Lizarbe et al., 2013) and increased cerebral diffusion values in mid-term HFD-fed male mice in agreement with vasogenic inflammation (Guadilla et al., 2021). Applying magnetization transfer imaging (MTI) (Boer, 1995), higher cerebral MT ratios (MTR) were correlated with the amount of fat stores in adolescent humans, results that were claimed as consistent with adiposity-related alterations in the phospholipid composition of cerebral lipids (Schwartz et al., 2014). MRS findings include increased HFD-induced concentrations of surrogate markers of inflammation, glucose and lipid metabolism (Lizarbe et al., 2018, 2019). Again, many of the mentioned pre-clinical studies were performed using male mice exclusively, with the particular response of females to HFD intake, remaining to be characterized. Moreover, the specific temporal sequence of the cerebral inflammatory and metabolic changes underlying obesity development in both sexes remained to be defined.

On these grounds, we sought here to investigate, systematically, the sexual differences in cerebral alterations underlying obesity development in mice using MRI and MRS. We hypothesized that MR methodologies could be used to follow the early HFD-induced microstructural rearrangements detected previously *ex vivo*, and quantify robustly, their further progression to the obese phenotype *in vivo*. Moreover, since sex is known to exert a critical influence on the HFDs response, we suspected that it could lead to different temporal MRI patterns and metabolic profiles in males and females. To address this, we designed a physiological and *multiparametric* MRI pipeline to investigate the effects of diet, time, and sex in the mouse brain. Particularly, we examined the T_2_, MTR, and DWI profiles in four cerebral areas related to homeostatic and non-homeostatic control of food intake: the hypothalamus, the hippocampus, the NAc, and the ILA, and tested, using a linear mixed estimation (LME) model, if diet, time and area where significantly determining the MRI parameters. We additionally obtained the regional neurochemical profiles using ^1^H HRMAS, providing an integral analysis of the metabolic and inflammatory responses to obesity, in the brain of both, male and female mice.

## Materials and methods

### Experimental models

All experimental procedures studies were approved by the Ethic Committees of the Biomedical Research Institute ‘‘Alberto Sols,’’ CSIC and the Community of Madrid (PROEX 124/15) and follow the national (R.D.53/2013) and European Community guidelines (2010/62/UE) for care and management of experimental animals. Mice were housed in the animal premises of our institution (Reg. No. ES280790000188) and cared for by specialized personnel. Design, implementation, and reporting adhere to the ARRIVE guidelines. Samples sizes were estimated using G*power statistical software^1^ to obtain at least a *p* < 0.05, in three (simplified) scenarios: ANOVA within-factors repeated measures to estimate the effects of time on the MR parameters of a specific group (α = 0.05, effect size = 0.35, number of measurements = 5, correlation = 0.8), ANOVA within-between interactions to account for the time-diet effects (α = 0.05, effect size = 0.25, number of measurements = 5, correlation = 0.8) or time*area-diet factors (α = 0.05, effect size = 0.15, number of measurements = 5 × 4, correlation = 0.8). The resulting n per group were 8, 7, and 8; and the final n = 8 was chosen as a “consensus” value in the three cases simulated.

MRI studies were carried out in adult male (*n* = 15, 25.5 ± 2g) and female (*n* = 15, 18.7 ± 2g) C57BL/6 mice (9–12 weeks-old), housed in cages of 3-5 animals in a room with controlled temperature (22°C) and humidity (45%) with a 12-h-light/12-h-dark cycle. All animals were initially provided with *ad libitum* access to drinking water and standard laboratory diet (SD) chow (9% fat, 71% carbohydrates, 20% proteins, 3.15 kcal/g; A04-10 U8221G10R, SAFE) (male mice) or (13% fat, 67% carbohydrates, 20% proteins, 2.9 Kcal/g; Teklad) (female mice). Subsequently, mice were randomly divided into two cohorts, the HFD group (8 males and 8 females) fed with a 60% HFD (butter-based) (60% fat, 25% carbohydrates, 15% proteins, 5.51 kcal/g, U8978P version 0019; SAFE) while the control group (*n* = 7 males and 7 females) continued with the SD. MRI studies took place before diet diversification (*t* = 0) and on days 7, 14, 28, and 10 weeks after diet onset. Body weight and temperature, blood glucose levels, and food intake were controlled on a weekly basis. Neither randomization nor blinding methods were applied. HRMAS metabolic analysis of brain samples was performed in four additional animal cohorts, *n* = 14 HFD and *n* = 14 controls (*n* = 7 males and *n* = 7 females for each subset), under the same housing and diet conditions described above.

### Animal preparation for magnetic resonance imaging

MRI procedures were carried out between 8:30 a.m and 12:30 p.m., during the light period, and in fed *ad libitum* conditions. Only 3 animals were imaged per experimental session, to minimize variability due to changes in the circadian rhythms. Mice were anesthetized in an individual acrylic induction chamber with 3% isoflurane/oxygen for 3 min for induction and remained with 2% for four additional minutes in order to reach a steady condition. Once stabilized, mice were transferred and immobilized in a semi-cylindrical home-built acrylic holder with a bite bar coupled to a nose cone mask to administer anesthesia during the imaging time. Before inserting animals into the scanner, blood glucose levels were measured from a drop of blood from the tip of the tail, using a standard glucometer (Accu-Chek^®^Aviva, Roche), under anesthesia. Throughout the course of imaging acquisitions, isoflurane levels were kept between 1 and 1.5% to maintain the breathing rhythm between 40 and 70 breaths per minute. Respiration rate was monitored using a sensor located below the abdomen, and body temperature through a rectal probe with a Biotrig physiological monitor (SA Instruments, Inc. NY, USA). To avoid a decrease in animal temperature during MRI acquisition, the holder was covered with a circulating heated water blanket.

### Magnetic resonance imaging system and sequences

MRI experiments were performed on a 7 Tesla horizontal-bore (16 cm diameter) superconducting magnet equipped with a ^1^H selective birdcage resonator of 23 mm and a 90 mm diameter gradient insert (360 mT/m) (Biospec^®^ 7T, Bruker Biospin, Ettlingen, Germany). Imaging data were acquired using a Hewlett-Packard console running Paravision 5.1 or 6.1 software (Bruker Medical Gmbh, Ettlingen, Germany) for males and females, respectively, operating under a Linux environment.

Localization of the regions of interest (ROIs) -hypothalamus, hippocampus, NAc, and ILA- was achieved by performing axial T_2_ weighted spin echo anatomical images with field of view (FOV) = 21 × 21 mm^2^ and in-plane resolution of 82 × 82 μm^2^. A rapid acquisition with relaxation enhancement (RARE) sequence was performed with a repetition time (TR) = 2500 ms, echo time (TE) = 60 ms, RARE factor = 8, number of averages (Av) = 1, number of slices = 5 in axial orientation, slice thickness = 1.25 mm. The resulting images were compared to an anatomical atlas (Garcia-Serrano et al., 2022) and the ROIs were determined for every animal. T_2_, apparent diffusion coefficient (ADC), and MTR maps acquisition were performed across the axial planes containing the mentioned ROIs, with one axial slice covering the hypothalamus and hippocampal regions, and another comprising the NAc and ILA ROIs (FOV = 21 × 21 mm^2^, Mtx = 128 × 128, corresponding to an in-plane resolution of 164 × 164 μm^2^, slice thickness = 1.25 mm).

The acquisition of T_2_ maps relied on a set of T_2_W images acquired with TR = 5000 ms, TE = 12 ms, Av = 1, and 50 different TE values (12 ms < TE < 600 ms). The set of DWI was acquired with the diffusion gradients applied along three orthogonal directions: left–right, antero-posterior and head–feet, with TR = 3000 ms, TE = 31 ms, four-shot EPI readout, Av = 3, diffusion gradient separation (Δ) = 20 ms and duration (δ) = 4 ms. A collection of nine b-values [*b* = γ^2^δ^2^*G*^2^(Δ−δ/3), where γ is the gyromagnetic ratio of the proton and G is diffusion gradients strength] was used (200 < b < 2000 smm^–2^) during the experiments with males, while a shorter sequence with 3 b-values (300 < b < 1200 smm^–2^) during acquisition for females. For MTR, two sets of images were acquired. The first one was obtained with a sequence including a saturation pulse intended to transfer magnetization from the bound-water pool (MT on) to the free-water molecules, employing a radiofrequency pulse train (*N* = 50) of bandwidth = 550 Hz, length = 5 ms, power = 5.5 μT and offset = 1,500 Hz. The second set was imaged without the saturation pulse (MT off). Acquisition parameters were: TR = 2500 ms, TE = 9.8 ms, and Av = 1.

### Image analysis

T_2_, ADC, and MTR maps were generated using an in-house program developed on Matlab v7 (The Mathworks, Nattick, MA, USA), performing an automatic –and blinded- processing of the images, generating parametric images by fitting the MR signals to the appropriate mathematical equation. Briefly, the T2 maps were obtained by fitting, pixel-by-pixel, the set of signal intensities in T_2_W images and the corresponding echo times to a monoexponential decay *S*(*TE*) = *S*_0_*e*^−*TE*/*T*_2_^, where S_0_ represents the signal intensity at TE∼0. ADC maps were, likewise, obtained after fitting each pixel signal from DWI to a monoexponential decay, $\frac{S\,\left(b\right)}{S_{0}}=e^{-b\cdot A\,D\,C},$ relating the MRI signal loss *S(b)*, with the ADC, with S_0_ representing the signal value in the absence of diffusion gradients. MTR maps were computed by digitally subtracting the two sets of images obtained (MT pulse *on* or MT pulse *off*). For each voxel, we obtained a corresponding MTR value $\left(M\,T\,R=\frac{\left(S_{0}-S_{M\,T}\right)}{S_{0}}\cdot 100\right)$, where S_0_ is the magnitude of the tissue signal without the MT pulse, and *S*_*MT*_ corresponds to the MT *on* signal.

The four ROIs investigated were manually delimited for each animal on the parametric maps, with the help of the high resolution (82 × 82 μm^2^) T_2_WI and the brain anatomical atlas (Paxinos and Franklin, 2001) as references. Briefly, the hypothalamus was selected as a 70 voxels rectangular region, the hippocampus as two squared 30 voxels regions, the NAc as two oval 40-voxels regions, and the ILA as a circular 20 voxels region (rough values). For every animal, values of the parametric maps were automatically filtered to remove values of the cerebrospinal fluid (CSF)-containing voxels or potential artifacts, with T_2_ and MTR maps being filtered as 30 < T2 < 80 ms and MTR > 0, and DWI images restricted to 0 < ADC < 1200 μm^2^/s. Subsequently, average values per ROI and animal were calculated. For each diet group, time point, and region, values falling outside the (1^st^/3th quartile ± 1.5*interquartile range) were considered outliers and removed from calculations.

### High-resolution magic angle spinning

Prior to the *ex vivo* HRMAS acquisitions, animals were euthanized under anesthesia using a high-power (5kW) microwave (Microwave Fixation System TMW-4012C, Muromachi Kikai Co., Ltd, Japan) focused on the brain. This method allows for arresting rapidly the cerebral metabolism by heat inactivation (O’Callaghan and Sriram, 2004). Subsequently, fixed brains were removed from the skull, and tissue from the different brain areas was dissected: the hypothalamus, hippocampus, and regions corresponding to the NAc and ILA. Samples were stored and preserved at –80°C until their analysis with HRMAS spectroscopy. Briefly, a small piece (10–15 mg) of the frozen sample was introduced in a zirconium rotor (4mm of outside diameter) and filled with D_2_O up to 50 μL. Then, the rotor was transferred into the HRMAS probe pre-cooled at 4°C. ^1^H HRMAS spectra were acquired on an 11.7T MHz Bruker AVANCE Spectrometer operating at 500.13 MHz, 5 kHz spinning rate, and a temperature of 4°C. Carr-Purcell-Meiboom-Gill protocol was employed with 5 s of relaxation delay, 2 s of water suppression delay, a total TE of 36 ms, 32K data points, and 128 scans. Spectra were processed with LCModel (Provencher, 2001), which allows the quantification of detectable metabolites in the cerebral areas investigated. This software fits each spectrum as a linear combination of brain metabolites’ prototypical spectra contained in a homemade data base (Righi et al., 2018), and calculates values of the metabolic concentrations and estimated percentage of standard deviation. Our data base of cerebral metabolites included acetate, alanine, aspartate, choline (Cho), creatine (Cr), GABA, glucose (Glc), glutamine (Gln), glutamate (Glu), glycine (Gly), glycerylphosphorylcholine (GPC), lactate (Lac), myo-inositol (mI), N-acetyl-aspartate (NAA), phosphocholine (PCh), phosphocreatine (PCr), taurine (Tau), signals from mobile lipids such as Lip13a, Lip09, Lip20, and macromoelcules (M14), and the corresponding sums Cho+GPC+PCh, mI+Gly, Cr+PCr, Glu+Gln, mm14+Lip13a+Lip13b, among others. Metabolite concentrations are expressed as the ratios to the total creatine content (Cr+PCr signal).

### Statistical analysis

Changes in the physiologic measurements were assessed using GraphPad Prism 8.0, (GraphPad Software, Inc., La Jolla, CA). Time-course of blood glucose concentration for each diet group (separately) was evaluated with a repeated-measures one-way ANOVA test, corrected for multiple comparisons (Bonferroni’s multiple comparisons test). Differences in food intake and body weight between diet cohorts were investigated using multiple *t*-tests (one for each time point), corrected for multiple comparisons using the Holm-Sidak method.

Statistical analyses of the MR data were performed using the R software (R Core Team, 2020). Since our experimental design involved different types of variables, including *fixed* effects -variables that are constant across individuals, such as diet or time points-, *nested* data -multiple observations within a particular subject, such as the four brain regions assessed-, and *random components* - fluctuations incorporated by each subject- to be investigated, a statistical approach that could consider all the variables were adopted, and linear mixed-effects models were designed. Briefly, LME models include the abovementioned fixed and random-effects terms in a linear predictor expression, from which a mean response variable (or “Y”) for each predictor (or “X”) is evaluated (Bates et al., 2015). LME provides flexible frameworks for the analysis of longitudinal data, and have demonstrated superior statistical power in identifying longitudinal group changes than other assessments, such as repeated measures ANOVA (Bernal-Rusiel et al., 2013), as well as remarkable robustness again mild violations of model assumptions (Schielzeth et al., 2020).

In our study, the T_2_, ADC, and MTR cerebral values (our response variables) were fitted to a variety of LME regression models, using the “lme” function of “nlme” package (Pinheiro et al., 2022), including *diet, time*, and *region* as fixed effects, or predictor variables, and time over area and mouse as random effects with an autoregressive structure (Fox and Weisberg, 2019). Regressions were performed using a multilevel strategy, which is based on fitting several models -from the simplest, with no fixed effects, only the random components (time point/area/animal), to the most complicated, which included all predictor variables and their corresponding interactions- to subsequently choose the best model, according to the *Akaike’s information criterion* (AIC), a goodness-of-fit measure that is corrected for model complexity (Fox and Weisberg, 2019). The following models were designed: the “baseline” model (“B”), with only the random components; the area model (“A”), which included area as a fixed effect; the time model (“AT”), which added time as fixed effect; time random slope (“ATrs”), which assumed that the response variable Y could vary between time-points in a subject-dependent manner; diet (“ATrsD”), adding the type of diet as a fixed effect; the interaction time:area (“ATrsD.TA”), which included the fact that depending on the area measured, Y could change differently between times; interaction diet:time (“ATrsD.TA.DT”), to test if the type of diet-induced different time-changes on Y; and diet:area (“ATrsD.TA.DT.DA”) and diet:area:time (“ATrsD.TA.DT.DA.DAT”) models, which included the corresponding interactions. By construction, each new model contained the effects of the previous plus the corresponding new addition. The model with the lowest AIC was identified automatically using the “stepAIC” function of the “MASS” package (Venables and Ripley, 2002). Once the best model was chosen, type III ANOVA tests were performed with the “Anova” function of the “car” package (Fox and Weisberg, 2019), to assess the relevance of each effect or interaction, as well as the potential difference between baseline and the 7, 14, 28, and 70 days of diet. Such time-level contrasts were performed for each diet cohort and area independently using the “emmeans” package (Lenth, 2022) and a Dunnett correction for 4 multiple comparisons. The main model assumptions for LME approaches, which are based on checking if residuals and random effect coefficients are independent and identically distributed (Schielzeth et al., 2020), were examined in each case by plotting the standardized residuals vs. the fitted values or vs. the quantiles of the normal distribution, using the “qqPlot” function of the “nlme” package. All data was log-transformed prior lme fitting to improve the normality distribution of the residuals, and regional outliers were removed.

Metabolite concentration from the HRMAS data was also fitted to different linear mixed regression models using R, with diet and area, and their corresponding interaction, as fixed effects, and area/mouse as the random components. Similarly to the MRI data, we fitted sequentially different models -from the simplest to the most complex- and assessed the potential improvement in the metabolites’ concentration estimation using ANOVA tests between models. Afterward we performed Type III ANOVA tests of the selected model and assessed the specific effects of diet and area for each sex cohort metabolite.

## Results

### Male mice develop faster an obese phenotype

**FIGURE 1:**
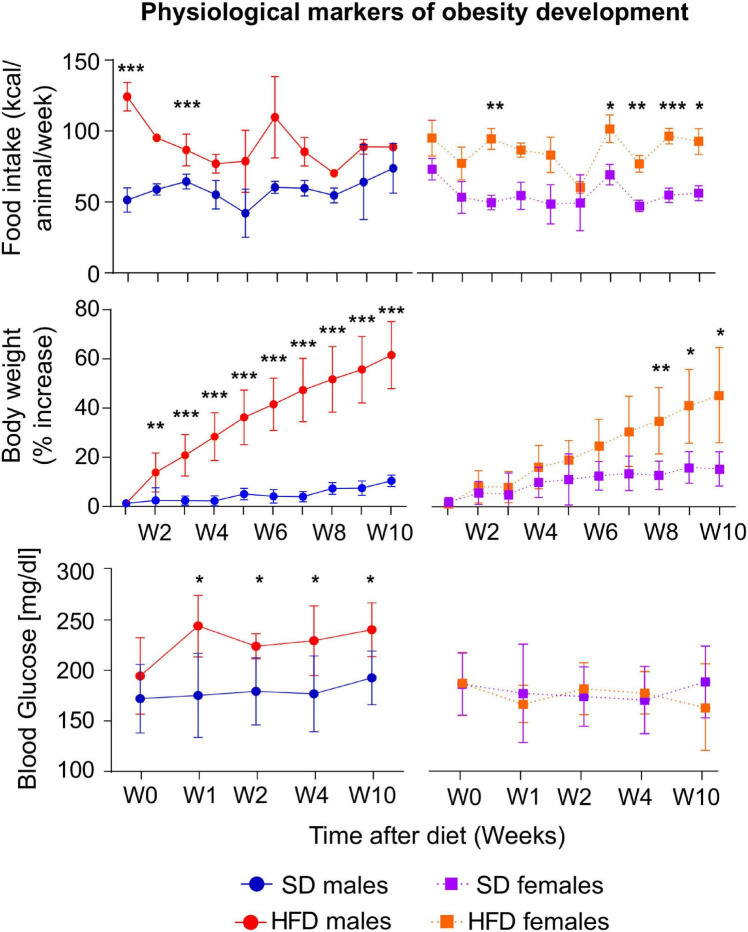
Physiological changes during HFD or SD consumption. kcal/animal/week consumed (top), percentage of body weight changes (center), and blood glucose (bottom), on males (left) and females (right) for the HFD or SD cohorts, measured either weekly from week (W) 1 to week 10 from diet diversification, or measured before the MRI sessions in non-fasting conditions, for the blood glucose values. All values are expressed as (mean ± stdev) (*p < 0.05, **p < 0.01, ***p < 0.001).

Male and female mice from the HFD cohorts consumed more calories than their respective counter-mates (Figure 1 top). Those differences were statistically significant for male mice during week one W1 and W2, (left panel) while, in females, remarkable changes occurred during W3, W7, W8, W9, and W10 (right panel). Increased consumption resulted in higher body weights of HFD mice, being statistically significant from day 14 (D14) on males, and only from W8 on females (Figure 1 center). Moreover, male animals increased their BW by approximately 60%, while females augmented around 40%, as compared to basal measurements. Blood glucose tests revealed increased values of the HFD cohort after 7 days of diet diversification, only in males, while females depicted steady concentrations during the 10 weeks of high-fat consumption (Figure 1 bottom).

### Multiparametric brain magnetic resonance imaging follow-up of mice consuming high-fat diet or standard laboratory diet

**FIGURE 2:**
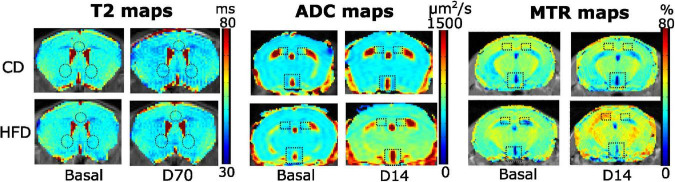
Representative T2, ADC and MTR maps of control (top) or HFD-fed (bottom) male animals in different time points. T2 maps illustrate the T2 values of male animals in the brain slice containing the NAc (bottom circles) and ILA (top circle) regions, on basal (left) or D70 measurements. A slight shift to darker values can be appreciated in the D70 maps, both in SD and HFD mice. ADC maps show the corresponding coefficients of a male mouse in the slice containing the hypothalamus (bottom rectangle) or hippocampus (top squares), on the basal and D14 time points. A change to yellower/reddish colors can be seen in the HFD-D14 map. MTR plots depict the basal (left) and D14 (right) values on a male animal under SD (top), or HFD (bottom), on the slice containing the hypothalamus and hippocampus, with the corresponding ROIs. The HFD mouse images at day 14 have higher MTR values, and are slightly shifted to yellow-red colors. All maps are depicted for illustrative purposes only.

By fitting the MRI signals to the corresponding T_2_, ADC, and MTR equations, we obtained parametric maps for the brain of each animal, day, and area of interest. Individual maps from representative animals are shown in Figure 2, where differences in color intensity suggest underlying differences between SD (top) or HFD (bottom) animals in the time-points shown.

**FIGURE 3:**
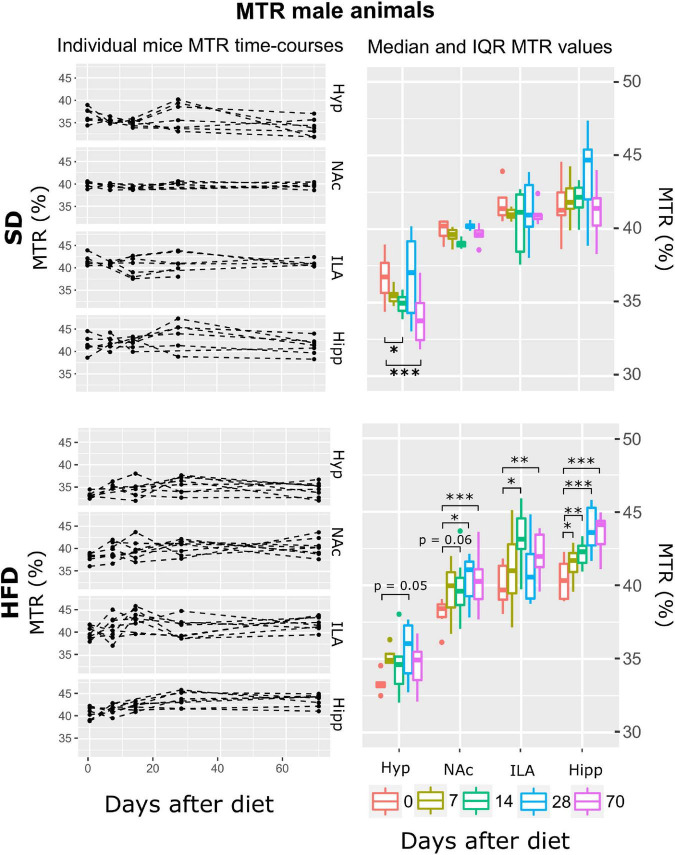
MTR changes on male mice during HFD or SD consumption. MTR evolution on SD-fed (top panels) or HFD (bottom panels) male mice in the four cerebral regions investigated: hypothalamus (Hyp), nucleus accumbens (NAc), ipsilateral area (ILA) and hippocampus (Hipp) at all time points measured. Left panels depict the individual MTR regional values at each time point, with dashed lines connecting the neighboring points. Right boards illustrate box plots of MTR vs. time-point measurements for the 4 regions assessed, with each color representing the different time-points evaluated, expressed as days after diet diversification. Note how MTR increases with time only on animals under HFD (*p < 0.05, **p < 0.01, ***p < 0.001).

**FIGURE 4:**
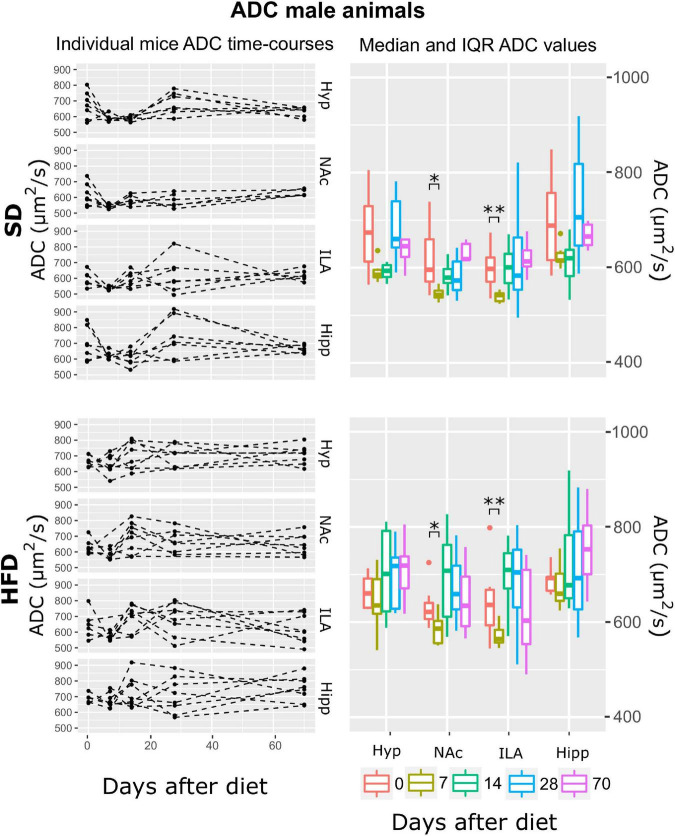
ADC changes on male mice during HFD or SD consumption. ADC evolution on SD-fed (top panels) or HFD (bottom panels) male mice in the four brain areas assessed: hypothalamus (Hyp), nucleus accumbens (NAc), ipsilateral area (ILA) and hippocampus (Hipp) at all time points measured. Left panels depict the individual regional values at each time point, with dashed lines connecting the neighboring points. Right boards illustrate box plots of ADC vs. time-point for the 4 regions assessed, with each color representing the specific time-point evaluated, as days after diet diversification (*p < 0.05, **p < 0.01).

**FIGURE 5:**
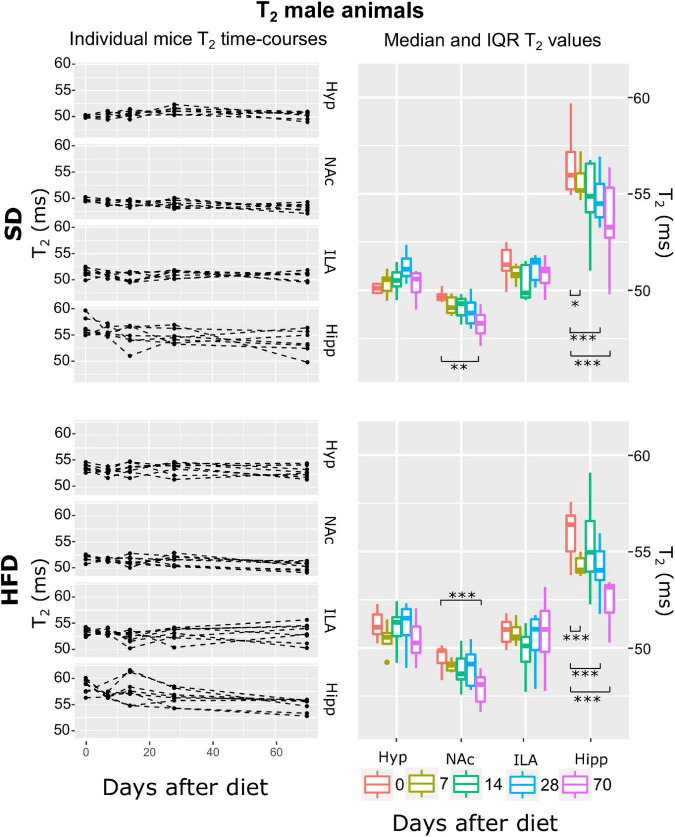
T2 changes on male mice during HFD or SD consumption. T2 changes on SD-fed (top panels) or HFD (bottom panels) male mice in the four regions investigated: hypothalamus (Hyp), nucleus accumbens (NAc), ipsilateral area (ILA) and hippocampus (Hipp) at all time points measured. Left panels depict the individual T2 regional values at each time point, with dashed lines connecting the adjacent points. Right panels illustrate box plots of T2 vs. time-point for the 4 areas, with each color representing the specific time-point, as days after diet diversification. Note how T2 behaves similarly under both diets, with decreasing values on the NAc and hippocampus (*p < 0.05, **p < 0.01, ***p < 0.001).

**FIGURE 6:**
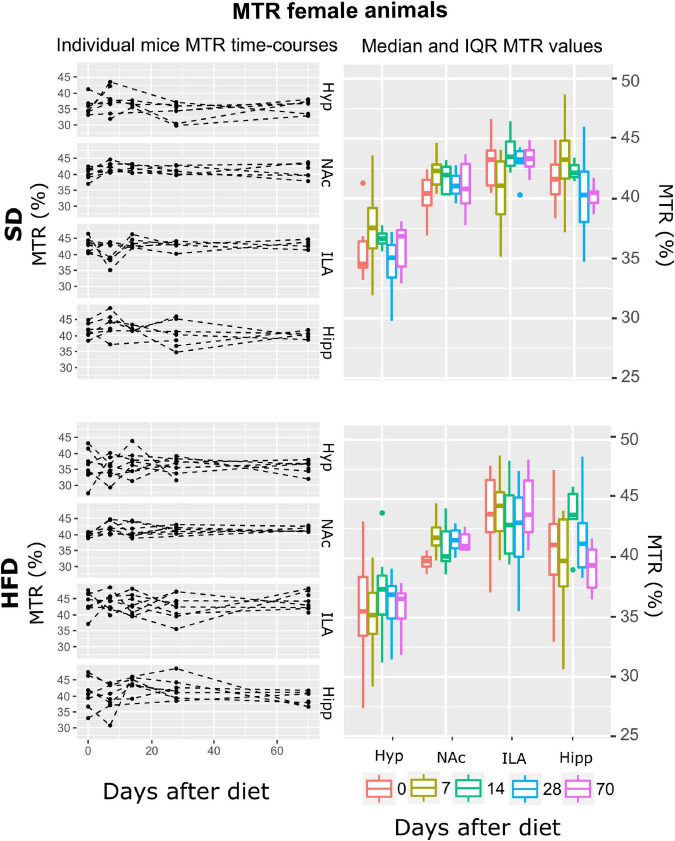
MTR changes on female mice during HFD or SD consumption. MTR values on SD-fed (top boards) or HFD (bottom boards) female animals in the four cerebral regions investigated: hypothalamus (Hyp), nucleus accumbens (NAc), ipsilateral area (ILA) and hippocampus (Hipp) at all time points measured. Left panels depict the individual MTR regional values at each time point, with dashed lines connecting the adjoining points. Right panels show box plots of MTR vs. time-points for the 4 regions evaluated, with each color representing the specific time-point, as days after diet diversification. Note how MTR does not depict remarkable changes with time or diet.

**FIGURE 7:**
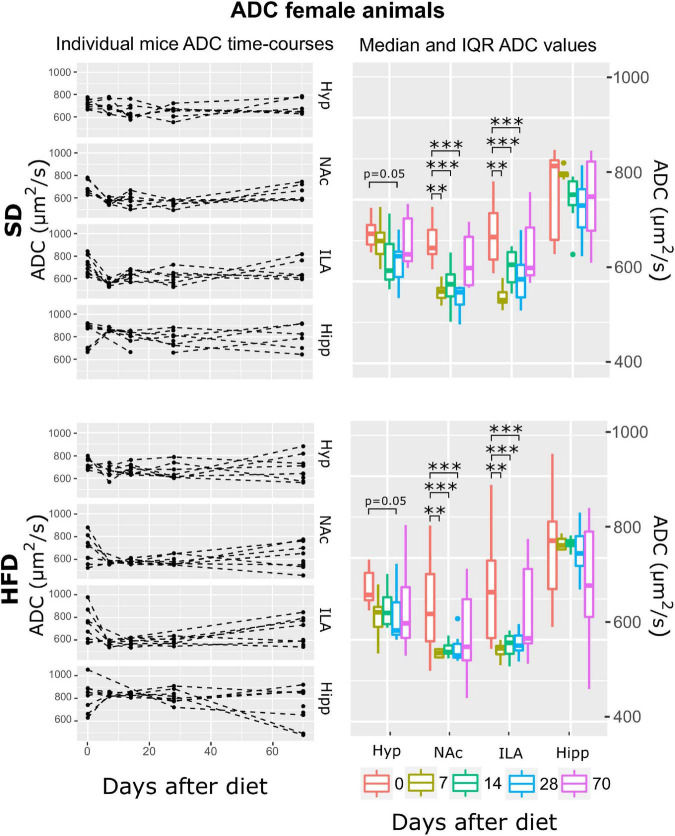
ADC changes on female mice during HFD or SD consumption. ADC time-evolution on SD-fed (top panels) or HFD (bottom panels) female mice in the four brain areas assessed: hypothalamus (Hyp), nucleus accumbens (NAc), ipsilateral area (ILA) and hippocampus (Hipp) at all time points measured. Left panels depict the individual regional values at each time point, with dashed lines connecting the neighboring points. Right panels depict box plots of ADC vs. time for the 4 regions studied, with each color corresponding to the same time-point, in days after diet diversification. Note how ADC decreases during the first measurements under both diets (**p < 0.01, ***p < 0.001).

**FIGURE 8:**
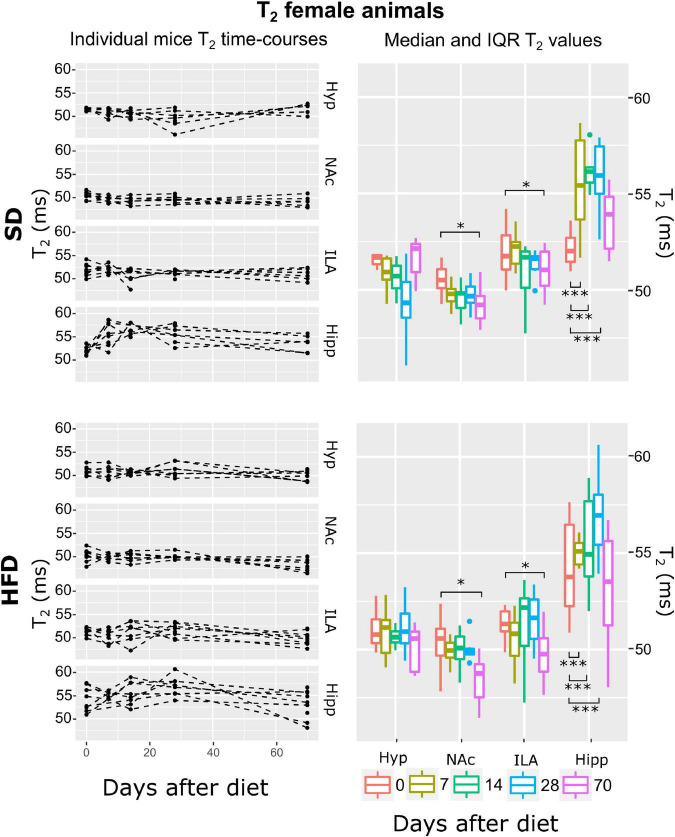
T2 changes on female mice during HFD or SD consumption. T2 changes on SD-fed (top panels) or HFD (bottom panels) female mice in the four regions investigated: hypothalamus (Hyp), nucleus accumbens (NAc), ipsilateral area (ILA) and hippocampus (Hipp) at all time points measured. Left panels depict the individual T2 regional values at each time point, with dashed lines connecting the adjacent points. Right panels illustrate box plots of T2 vs. time-point for the 4 areas, with each color representing the specific time-point, as days after diet diversification. Note how T2 behaves similarly under both diets, with decreasing values on the NA0c and ILA, and increasing on the hippocampus (*p < 0.05, ***p < 0.001).

Subsequently, and for each animal, mean T_2_, ADC, and MTR individual regional values were calculated for each time-point-after-diet MRI measurement. The graphical representation of the individual animal time courses, displayed by separating the diet cohorts into separated panels, allowed an initial qualitative assessment of the respective time-course changes and animal variability (Figures 3–8, left panels). Box plot representations of the MR parameters vs. time, which include median values and their interquartile ranges (IQR), allowed the visualization o potential MR changes between time points for each diet and sex cohort (Figures 3–8, right panels).

After the initial visual qualitative assessment of the brain MRI parameters, six parallel multi-level lme fittings were performed (one for each MRI parameter, two sexes), and the corresponding best model was selected using the AIC criteria. Graphical validation of the corresponding best models (plots not shown) achieved satisfactory results, in terms of residual homogeneity and normality. The following “ Male mice exhibit increasing MTR and elevated ADC during HFD consumption; both diet cohorts reflect decreasing ADC the first fortnight and decreased T2 at longer periods.” and “ Female mice under both diets display no changes on MTR, decreases of ADC, and T2 decreases in all areas except in the hippocampus during HFD” detail the main findings provided by the lme approach, from males and females, respectively.

#### Male mice exhibit increasing magnetization transfer ratio and elevated apparent diffusion coefficient during high-fat diet consumption; both diet cohorts reflect decreasing apparent diffusion coefficient the first fortnight and decreased T2 at longer periods

The model that best fitted the MTR data included area, time, diet, and their double interactions (ATrsD.AT.DT.DA model). Subsequent type III ANOVA tests of such a model showed significant effects of all predictors and interactions (*p* < 0.001) (Table 1). Post-hoc analysis to assess the difference between measurements at day 0 and the rest of the time points, revealed that animals under SD animals, showed decreased MTR values in the hypothalamus 14 and 70 days after baseline measurements (p < 0.05 and 0.001, respectively), and no changes in the NAc, ILA or hippocampus during the time of the study (Figure 3, top panels). HFD mice showed a tendency to increase MTR in the hypothalamus after 28 days of diet (*p* = 0.05), that MTR increased significantly on the NAc (*p* = 0. 06, *p* < 0.05, and *p* < 0.001, for days 14, 28 and 70), ILA (*p* < 0.05, *p* < 0.005) for days 14 and 70, and the hippocampus (*p* < 0.05, *p* < 0.005, *p* < 0.001, *p* < 0.0001) for the 7, 14, 28 and 70 days of the diet, respectively (Figure 3, bottom panels).

**TABLE 1:** Selected “best model” and results of the Anova Type III tests of the predictor variables on log-transformed MTR, ADC and T2 data of male (m) (top) and female (f) (bottom) mice.

	Param.	Model/Effects	A	T	D	AT	DA	TD	DAT
m	MTR	ATrsD.AT.DT.DA	*p* < 0.001	*p* < 0.001	*p* < 0.001	*p* < 0.001	*p* < 0.001	*p* < 0.001	n.i.
	ADC	ATrsD.AT	*p* < 0.001	*p* < 0.001	p < 0.001	*p* < 0.005	n.i.	n.i.	n.i.
	T2	ATrsD.AT.DT.DA	*p* < 0.001	n.s	n.s	*p* < 0.001	*p* < 0.001	n.s	n.i.
f	MTR	A	*p* < 0.001	n.i.	n.i.	n.i.	n.i.	n.i.	n.i.
	ADC	ATrsD.AT	*p* < 0.001	n.s.	n.s.	*p* < 0.001	n.i.	n.i.	n.i.
	T2	ATrsD.AT.DA	*p* < 0.001	n.s	n.s	*p* < 0.001	*p* < 0.05	n.s.	n.i.

Small *p*-values indicate significant effects of the fixed effects (A: Area; T: Time; D: Diet; AT: Area*time interaction; TD: Time*diet interaction; DA: Diet*area interaction; DAT; Diet*time*area interaction) on the corresponding MRI parameter. The “best model” used is specified in the third column (A: Area model; ATrsD.AT.DT.DA: Area, Time random slope, diet, area*time, diet*time, diet*area model, ATrsD.AT: Area, Time random slope, diet, area*time model).

Results for the ADC data showed that the best model that fitted the log (ADC) values included the fixed effects of time, area, diet, and the time:area interaction (ATrsD.AT model). Corresponding Type III tests depicted very remarkable effects of time, area, diet, and the area*time interaction (all *p* < 0.001) (Table 1). The significant effect of diet can be seen in Figure 4 as higher values of ADC of the HFD group in all areas and time points. Because there was no diet:time interaction effect, post-hoc analysis to test time points vs. baseline yielded the same differences between time points in both cohorts: decreased ADC values in the NaC and ILA 7 days after basal measurements (*p* < 0.05, *p* < 0.005, respectively).

Fitting the linear mixed-effect models to the log(T_2_) data and subsequent model-selection analysis revealed that the best model was the ATrsD.AT.DT.AT, which included all main effects and corresponding double interactions. Anova type III tests of the selected model indicated strong effects of the area alone, area:diet, and area:time interactions (*p* < 0.001) on T_2_, and no significant contributions of diet or time alone and the diet:time interaction (*p* > 0.05) (Table 1). *Post-hoc* tests between time points delivered similar results for both diet cohorts, with significant decreases of T_2_ over time in the NAc after 70 days of diet (*p* < 0.005 and *p* < 0.001) for SD and HFD mice, respectively, and generalized decreases in the hippocampus 7, 28 and 70 days after basal measurements (*p* < 0.01, *p* < 0.005, and *p* < 0.001) for SD and (*p* < 0.001) for HFD (Figure 5).

#### Female mice under both diets display no changes in magnetization transfer ratio, decreases of apparent diffusion coefficient, and T_2_ decreases in all areas except in the hippocampus during high-fat diet

From all the fitted regression models, the smaller AIC was reported on the A model, the one including only area as a fixed effect. Subsequent ANOVA tests showed a significant effect of area on MTR (*p* < 0.001) (Table 1). Because time was not included in the model, time *post-hoc* tests were not assessed. The independence of MTR of time or diet on females can be observed in Figure 6 as constant values along the two mentioned variables.

Apparent diffusion coefficient (ADC) regression modeling revealed the ATrsD.AT model is the one with the best fit. Corresponding Anova tests yielded very remarkable effects of time:area and area (*p* < 0.001) and no effects of diet or time alone, neither of their interaction (Table 1). *Post-hoc* tests revealed remarkable decreases of ADC in both cohorts, in the hypothalamus at 28 days (*p* = 0. 05) and in the NAc and ILA on days 7, 14, and 28 (*p* < 0.001 all) (Figure 7).

The multilevel lme strategy revealed that the best fit to the log-transformed T_2_ female data was the ATrsD.AT.DA which included all main effects and the area:time and diet:area interactions. Corresponding Anova type III tests model indicated strong effects of the area and area:time interaction (*p* < 0.001), a mild significant effect of area:diet (*p* < 0.05), and no significant contribution of diet or time alone (*p* > 0.05) (Table 1). *Post-hoc* tests showed that animals under both diets decreased T_2_ values on the last time point measurement in the NAc and ILA regions (*p* < 0.05). In the hippocampus, both animal cohorts showed increased T_2_ values, days 7, 14 and 28 (*p* < 0.001 all) (Figure 8).

### Cerebral metabolism is more altered in females than in males after 10 weeks of high-fat diet

**FIGURE 9:**
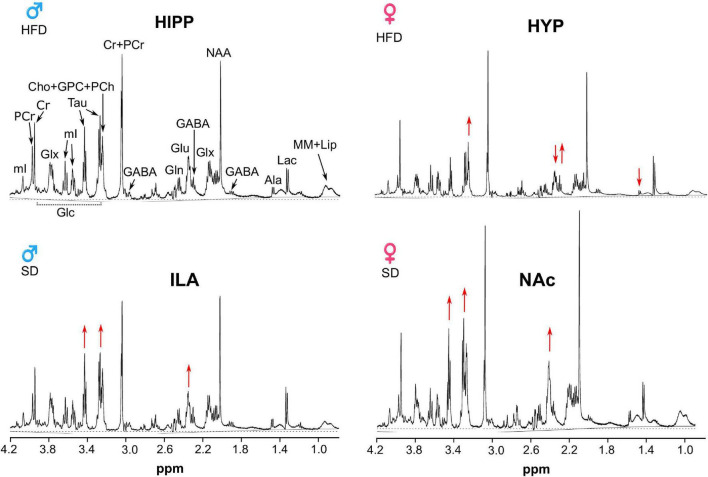
^1^H HRMAS spectra of representative female and male animals under SD or HFD. The hippocampus in a male HFD mouse (top left), hypothalamic region of a female HFD (top right), infralimbic area of a SD male (bottom left), nucleus accumbens (NAc) of a SD female (bottom right). Red arrows point the augmented or decreased signals characteristic of each region, such as the small Ala and Glu in the hypothalamus, or augmented Glu and Tau in the NAc or ILA.

High-resolution magic angle spinning (HRMAS) acquisition of the different samples resulted in very good quality spectra in almost all areas and animals, with a signal-to-noise ratio ranging from 19 to 26 and full width at half maximum linewidths around 2.5 Hz (Figure 9).

**FIGURE 10:**
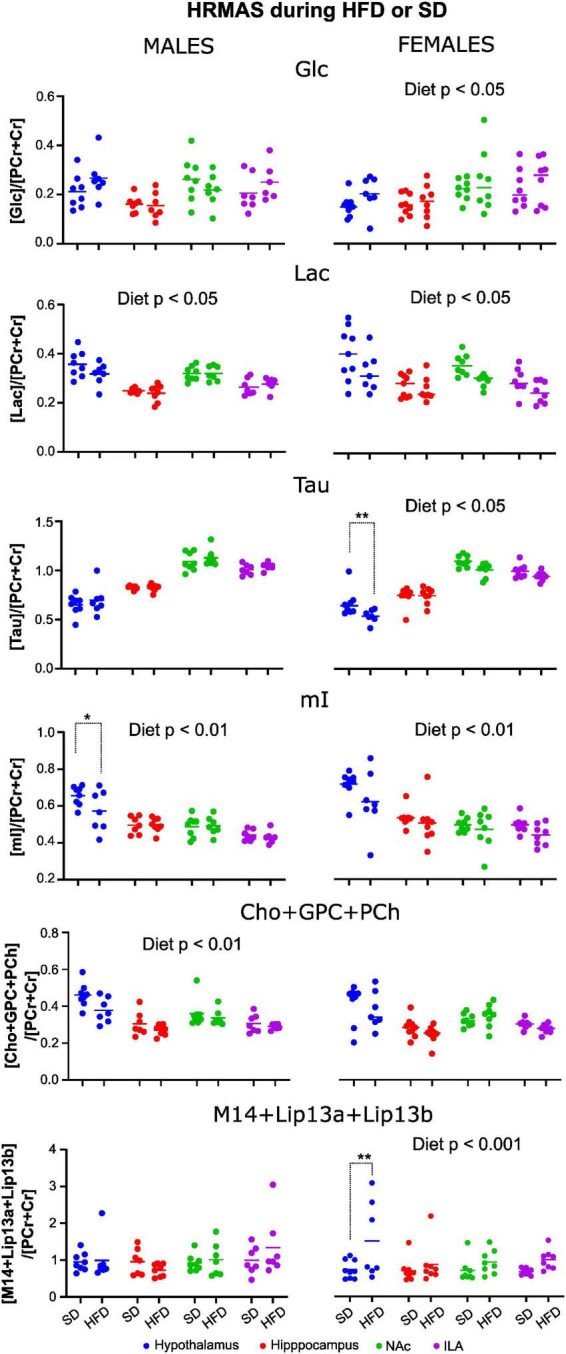
Relative metabolite concentrations after 10 weeks of HFD or SD consumption in male and female mice. Values of metabolite concentration of male (left) and female (right) animals, fed under a SD or HFD relative to the total creatine resonance. Dots represent the relative concentration obtained for each individual animal, and the continuous line represents de mean value within a diet group. Only those metabolites with significant diet effects are shown. (*p < 0.05, **p < 0.01).

Fitting of the spectra to the metabolic database yielded a good adjustment, and LCModel provided the relative concentration of metabolites for each sample. Metabolites with a standard deviation ≤ 20 were grouped per diet, region, and sex. Comparison of mean metabolite values from control or HFD cohorts showed that in male mice differences were small, while female mice tended to depict more altered metabolite concentrations with diet (Figure 10).

The model analysis revealed that the type of diet consumed significantly improved estimations of the relative concentration of GABA, Cho+GPC+PCh, Lac, and mI in males, and on GABA, Glc, Lac, mI, Tau, Lip13a, Lip20, and M14+Lip13 for females. Including the interaction diet:area in the model improved the estimations relevantly for GABA and mI in males, and for Tau, Lip13a, Lip20, and MM14 + lip13 in females, indicating that, for those metabolites, diet affected differently depending on the cerebral region investigated. Then, for each, metabolite, the most appropriate model (with or without interaction) was chosen, and Wald chi-square tests were performed to assess the impact of the fixed effects on the corresponding relative metabolite concentration (Table 2). Results depict remarkably lower concentrations of Lac, mI, and Tau, but higher Glc, during HFD consumption, in females (Figure 10, right panels). On male mice, we found remarkably lower concentrations of Cho+GPC+PCh and Lac during HFD (Figure 10, left panels). For those metabolites that showed significant area:diet interaction, *post-hoc* tests were performed for each area independently. Our results show that, from all areas, statistically remarkable changes were reported exclusively in the hypothalamus, with p-values on the rest of the regions above the significant threshold. Specifically, GABA and mI exhibited significantly lower hypothalamic concentrations during HFD on males (*p* < 0.05 both), while lip13a, lip20, and MM14 + lip13a remarkably increased concentration during HFD on females (*p* < 0.001, *p* < 0.005, *p* < 0.005), respectively.

**TABLE 2:** Results of the type III Anova tests of the fixed effects (diet and diet*area) on the HRMAS data, for male and female mice.

	Males		Females	
	Diet	Diet:area	Diet	Diet:area
Glc	n.s.	n.s.	*p* < 0.01	n.s.
Cho + gpc + pch	*p* < 0.01	n.s.	n.s.	n.s
Lac	*p* < 0.05	n.s.	*p* < 0.05	n.s.
mI	*p* < 0.01	*p* < 0.05	*p* < 0.05	n.s.
Tau	n.s.	n.s.	*p* < 0.01	*p* < 0.05
lip13a	n.s	n.s	*p* < 0.001	*p* < 0.05
lip20	n.s	n.s	*p* < 0.001	*p* < 0.01
mm14 + lip13	n.s	n.s	*p* < 0.001	*p* < 0.05

For simplicity, only those metabolites that had a significant (*p* < 0.05) diet effect on either male or female mice are shown. (n.s. non-significant).

## Discussion

In this work, we investigated longitudinally the progression of obesity in male and female mice, using MRI in four cerebral regions involved with appetite regulation and energy balance, and assessed the corresponding metabolic alterations after 10 weeks of HFD intake. We report that male mice fed with a high saturated-fat diet developed an obese phenotype accompanied by a notable diet-dependent increase in MTR, while females showed delayed obesity progress, did not depict alterations in blood glucose concentration, and experienced no differential *diet* or *time* effects on the cerebral MTR, despite the HFD-induced increased caloric intake. We also report ADC decreases in all cohorts, concentrated mainly during the first fortnight, and an association of HFD with higher ADCs in males that does not change with time. In both sexes and independently of the diet consumed, a remarkable long-term decrease in T_2_ values was observed in all regions, except in the female hippocampus. Physiologic and MR results are in agreement with preceding investigations reporting greater resistance to obesity development in females, potentially promoted by hormonal effects (Asarian and Geary, 2013). Moreover, the resulting MTR timeline on HFD or control male and female animals suggests that cerebral MTR values may provide a robust, non-invasive, biomarker of obesity development. The neurochemical profile, on the contrary, was more altered in HFD female animals after 10 weeks of diet.

### Sexual dimorphism in magnetic resonance imaging during high-fat diet

Magnetization transfer imaging enables the elucidation of the changes in the macromolecular environment of the biological tissues and has been predominantly used in the clinic as related to multiple sclerosis studies (Filippi and Agosta, 2007), while few imaging studies used it to investigate obesity-related changes (Kullmann et al., 2020). The statistical assessment of our data revealed a clear effect of diet on the MTR of male animals, as well that the time changes of MTR were also diet-dependent, and area-related. On males, the significant MTR increases during HFD, detected in all areas, and in the hippocampus as early as 7 days after diet diversification, are consistent with increases in macromolecule-bound water content. These results are in agreement with the augmented presence of astrocytes and microglia that occur during HFD-induced cerebral inflammation (Thaler et al., 2012; Xu et al., 2021). On females, on the contrary, neither diet nor any of its corresponding time or area interactions revealed altered MTR. These results suggest that female mice may not develop the same cerebral inflammatory process as males and are consistent with the lower physiological indicators of obesity.

Diffusion weighted imaging (DWI) studies use the water molecules’ Brownian diffusion as a surrogate marker of changes in tissue microstructure (Afzali et al., 2021). In biological tissues, water molecules diffuse among cellular membranes, macromolecules, vascular networks, and other components. Thus, potential physiological or pathological rearrangements are reflected in MRI-detectable changes in diffusion. Variations can include cellular swelling and shrinking events, vascular edemas, or changes in BBB permeability, and can differ between sub-regions or diffusion compartments (Le Bihan, 2013). Previous publications reported increases in cerebral diffusivity in DIO mouse models (Guadilla et al., 2021), and in patients with obesity (Alkan et al., 2008; Cazettes et al., 2011; Thomas et al., 2019). Notably, the authors explained the observed diffusion increases as linked to unspecific vascular edemas, but the causal mechanisms were not elucidated. In our experiments, we could detect remarkable effects of diet on the males’ ADC brain coefficients, with higher values on HFD mice, in agreement with the previous literature. Additionally, we found significant effects of time, observed in male cohorts as decreases during the first two weeks of measurements, and up to three weeks after basal images on females. During such a short period, animals were scanned at least three times, and we suspect that the detected changes can be related to the repeated scanning and the use of isoflurane, which has been associated to MRI-detectable changes in brain function and structure in mice (Bajwa et al., 2019; Tsurugizawa and Yoshimaru, 2021), and decreases in body temperature (Rufiange et al., 2020), which can affect ADC. We did not find specific time*diet or diet*area interactions, which implies similar ADC time-evolution in both cohorts and no remarkable diet-dependent regional differences in ADC.

T_2_ relaxation times characterize the transverse magnetization decay rates, because of the mutual interaction among neighboring protons. T_2_ differs between tissues, and it is also related to the macromolecular content, where slowly reorienting macromolecules induces dephasing between spins, promotes T_2_ relaxation, and results in shorter T_2_ values (Lizarbe et al., 2020). Examination of the T2 data revealed significant decreases with time, under both diets and sexes, except for the female hippocampus. The decrease in T_2_ is in agreement with the presence of macromolecules, which is consistent with our measurements of increased MTR on the HFD cohort. The fact that we detected the T_2_ decrease independently of the diet consumed, suggests additionally deteriorating mechanisms that are not detectable by MTR. Potentially, mechanisms similar to the known age-related loss of brain volume, which is accelerated by diabetes (Pell et al., 2012), take place during the 10 weeks of MRI measurements. The specific T_2_ increase of the female hippocampus could reflect a different process, suggesting increased free water content or alterations in the BBB permeability, and should be more carefully analyzed.

### Time effects of high-fat diet detected by magnetic resonance imaging

In the context of obesity development, two periods have been described, with the first phase of physiological adaptation to excessive caloric intake, and the second phase of more established obesity (Ziotopoulou et al., 2000; Benani et al., 2012). In our experiments, only one parameter, MTR, was sensible to the time*diet interaction, which means a different time evolution of MTR depending on the diet consumed. On male HFD mice, our data showed increasing MTR values with time that tended to establish in the last two time-point measurements, days 28 and 70 after diet diversification. These results suggest that MRI-detected changes may be more prominent during the abovementioned adaptative period, but further experiments should investigate the specific MTR evolution at longer periods of consumption.

### Sexual dimorphism in cerebral metabolic profiles under high-fat diet

Relative concentrations of brain metabolites after 10 weeks of HDF or SD consumption revealed stronger *diet* effects on females than on males, with several metabolites being remarkably affected by HFD consumption, prominently in the hypothalamus. Specifically, glucose and lipid content increased with HFD consumption, while Lac, Tau, myo-inositol, and the sum of choline compounds Cho+GPC+PCh decreased significantly in females, being most of the analogous comparisons in males below the threshold of statistical significance, except for Lac and the choline compounds.

Increased cerebral glucose values in females appeared without signs of altered blood glucose homeostasis, which might be reflecting decreased cerebral glucose utilization. This is consistent with the lower Lac levels detected in females, since lactate is one of the products of glucose metabolism (Mergenthaler et al., 2013). Dynamic measurements of glucose metabolism could confirm that trend (Duarte et al., 2012).

The lipid signals in a ^1^H HRMAS spectrum arise from protons of methyl and methylene groups of either long-chain saturated or unsaturated fatty acids, very small phospholipid nanovesicles, or even fast-tumbling triglyceride nanoparticles, with enough mobility to generate narrow resonances (Kaebisch et al., 2017). Our increased lipid *content* in the female metabolic profile compares well with an increased SFAs accumulation during HFD consumption exclusively in female animals, and remarkably in the hypothalamus. Notably, this is consistent with the fact that female mice contain larger number POMC neurons in the hypothalamus (Wang et al., 2018), as compared to males, potentially offering more SFA-binding sites (Jais and Bruning, 2017). Such increased lipid accumulation, however, does not trigger greater inflammatory responses, which has been related to estrogen signaling inducing anorexigenic effects (Zhu et al., 2015).

Taurine and myo-inositol, on the other hand, exert osmoregulatory roles that have been related to inflammation in the rodent brain (Duarte et al., 2012). In our study, relative levels of such osmolytes were decreased in female HFD animals. This describes a different alteration pattern of the neurochemical profile, as compared to other inflammatory diseases, potentially balancing the changes induced by altered glucose metabolism (Duarte et al., 2014).

Choline compounds are involved in cellular membrane metabolism, and increased turnover rates and cellular proliferation have been linked to augmented Cho resonance signal (Rae, 2014). Additionally, choline derivative compounds are responsible for membrane lipid synthesis and are precursors of the neurotransmitter acetylcholine (Afzali et al., 2021). In this sense, the relative decreases of Cho+GPC+PCh with HFD reported in this manuscript, in both sexes, are consistent with a shift of the equilibrium between membrane phospholipid anabolism to catabolism, suggesting either decreased membrane phospholipid turnover or increased synthesis. Notably, the increased synthesis compares well with the augmented MTR values on male animals.

### Linear mixed model-effects selection

The implementation of the multilevel strategy, combined with the AIC selection criteria and subsequent Anova tests, allowed us to obtain a semi-automatic method of fixed effects evaluation. Furthermore, we think that the possibility of presenting in an ordered manner the degree of influence of the different predictor variables (diet, time, area, and their interactions) on the MR parameters, for the different cohorts (Tables 1, 2), represents a very valuable visual integrative tool. For example, by looking at Table 1 it becomes clear that diet significantly affects only 2 parameters: MTR and ADC, and exclusively on male cohorts. The interaction of time with diet, on the other hand, was only remarkable in the MTR of male mice, which suggests that MTR may be a robust parameter to identify the effects of continuous HFD consumption, at least on male mice. Similar reasoning can help us point out that diet seems to affect the metabolic profile of female animals more, where additional regional differences are reported, as compared to males.

### Limitations

In this work, the experimental protocols for females or males involved two methodological differences that precluded the use of a joined statistical model. On one hand, the standard diet administrated to the male or female cohorts came from two different suppliers, involving a 4% change in the fat/carbohydrates total content, without affecting the percentage of saturated fat (0.6% in both cases). On the other hand, DWI sequences were acquired for male mice using 9 b-values (sequences lasting∼20 min), and only 3b for females (∼6 min duration). Thus, for male mice, fitting the DWI data profited from an increased number of b-values, but the longer acquisitions involved more chances of suffering from movement artifacts. Data analysis revealed that the number of pixels fitting the monoexponential model did not differ considerably between sexes, and we consequently estimated the two sequences as equivalent for the purpose of this study. However, to account for such methodological disparities, the statistical assessment was performed separately. Thus, while our study allowed us to observe a sexual disparity in the effects of HFD on cerebral MR parameters, future studies relying on exactly the same methodological conditions in both sexes need to be performed to provide quantifiable differences between sexes. On the other hand, our MRI results show only a few remarkable changes in the hypothalamus, while more changes were expected in this region. Future MRI sub-regionalization in hypothalamic nuclei may provide better results if analysis in the ARC nucleus becomes possible. Moreover, the extension of the current DWI approach into a *diffusion tensor* evaluation, including fractional anisotropy and mean diffusivity characterization, could provide additional information on the effects of diet in cerebral regional microstructure (Okudzhava et al., 2022). Finally, it should be noted that all metabolite values reported in this manuscript are referred to PCr+Cr concentration. Absolute quantification of HRMAS spectra delivers the most consistent information, however, this condition was not achievable in our system, and we thus opted for a relative quantification to total creatine content, which should not be altered during HFD feeding (Lizarbe et al., 2018), although a recent study found total creatine alterations under a high-fat high-sugar diet in some brain regions (Garcia-Serrano et al., 2022).

### Concluding remarks

In summary, our results demonstrate that consumption of SFA-enriched diets causes early changes in the brain that can be followed by MRI *in vivo* and quantified by HRMAS techniques, and that such alterations depend on sex. Findings are consistent with the appearance of an early inflammatory process in male mice that can be detected as MTR increases *in vivo*, most prominently in the hippocampus, while supporting the fact that this inflammation may not be taking place in females. After a long period of HFD consumption, decreased T_2_ measurements and decreased Cho+GPC+PCh relative concentrations suggest the presence of augmented macromolecular synthesis and content in male mice, specifically in the hypothalamus, which is consistent with long-term gliosis accumulation patterns. We found decreased ADC coefficients, potentially because of the repeated-measurements experimental design. Female mice did not express MRI-detectable diet changes in any of the brain areas related to appetite-control, showed a delayed obese phenotype development, but presented indicators of decreased cerebral glucose metabolism and increased lipid synthesis and accumulation, suggesting that HFD consumption alters cerebral metabolism but preserves better global energy homeostasis.

## Data availability statement

The data that support the findings of this study are available from the corresponding authors BL, blizarbe@iib.uam.es and PL-L, plopez@iib.uam.es, upon reasonable request.

## Ethics statement

This animal study was reviewed and approved by Ethic Committees of the Biomedical Research Institute “Alberto Sols”, CSIC and the Community of Madrid (PROEX 124/15).

## Author contributions

BWC: investigation, data curation, and formal analysis. DG: formal analysis, visualization, and data curation. SC: conceptualization, funding acquisition, resources, and writing –review and editing. PL-L: conceptualization, supervision, funding acquisition, and writing – review and editing. BL: conceptualization, formal analysis, software, visualization, writing – original draft, supervision, and project administration. All authors contributed to the article and approved the submitted version.

## Footnotes

1
http://www.gpower.hhu.de/

## Abbreviations

ADC: Apparent diffusion coefficient
ARC: Arcuate nucleus
BBB: Blood-brain barrier
Cho: Choline
Ctrl: Control
Cr: Creatine
DIO: Diet-induced obesity
DWI: Diffusion weighted imaging
TE: Echo time
GABA: glucose
Glc: Glucose
Glu: Glutamate
Gln: Glutamine
GPC: Glycerylphosphorylcholine
Gly: Glycine
HFD: High-fat diet
HRMAS: High-resolution magic angle spinning
Hipp: Hippocampus
Hyp: Hypothalamus
ILA: Infra limbic area
Lac: Lactate
LMM: Linear mixed model
MRI: Magnetic resonance imaging
MTI: Magnetization transfer imaging
MTR: Magnetization transfer ratio
MC: mesocorticolimbic complex
mI: Myo-inositol
NAA: N-acetyl -aspartate
NAc: Nucleus accumbens
n.s: Non-significant
PCh: Phosphocholine
PCr: Phosphocreatine
POMC: Proopiomelanocortin
ROI: Regions of interest
TR: Repetition time
RC: Reward centers
SFAs: Saturated fatty acids
SD: Standard laboratory diet
T_2_WI: T_2_ weighted imaging
Tau: Taurine
W: Week.
